# Functional Localization of Adult-Onset Idiopathic Nesidioblastosis

**DOI:** 10.1155/2022/2802975

**Published:** 2022-10-07

**Authors:** Jess C. Hercus, Pouneh Pasha, Sadiq Al Lawati, Peter Kim, Andre Mattman, Douglas Webber, David M. Thompson

**Affiliations:** ^1^Department of Biological Sciences, Simon Fraser University, Burnaby, British Columbia, Canada; ^2^Division of Internal Medicine, Department of Medicine, University of British Columbia, 5913-1081 Burrard St., Vancouver, British Columbia V6Z 1Y6, Canada; ^3^Division of Endocrinology, Department of Medicine, University of British Columbia, 2775 Laurel Street, 4th Floor, Vancouver, British Columbia V5Z 1M9, Canada; ^4^Division of General Surgery, Department of Medicine, University of British Columbia, 2775 Laurel Street, 11th Floor, Vancouver, British Columbia V5Z 1M9, Canada; ^5^Division of Pathology and Laboratory Medicine, Department of Medicine, University of British Columbia, G227-2211 Wesbrook Mall, Vancouver, British Columbia V6T 2B5, Canada

## Abstract

Nesidioblastosis is a rare pancreatic disorder involving enlarged beta cells throughout the pancreas, causing elevated insulin production. We present the case of a 53-year-old woman with the initial symptom of fasting hypoglycemia. No pancreatic lesions were indicated on computed tomography and magnetic resonance imaging scans, and an octreotide scan was negative for insulinoma. Selective arterial calcium stimulation (SACST) showed increased insulin production from the stimulation of 3 out of 5 arteries. The SACST results suggested a diagnosis of nesidioblastosis, which was confirmed by histopathology after a subtotal distal pancreatectomy. The patient has normal glucose tolerance after surgery with no further problems of hypoglycemia, indicating that this is a rare case of nesidioblastosis extending only partially through the pancreas.

## 1. Background

Nesidioblastosis is an extremely rare disorder; first described in infants in 1938 by Laidlaw [1], it was not found in adults until 1975 [2]. We present the first reported case of diffuse nesidioblastosis confined to the distal half of the pancreas. This case represents an intermediate form of nesidioblastosis, between highly localized focal nesidioblastosis and typical diffuse nesidioblastosis throughout the entire pancreas [3]. This distinction is clinically important as it will guide therapeutic decisions and likely prognosis for individual patients.

### 1.1. Case

A 53-year-old woman was referred to our clinic in 2017 with symptoms suggestive of hypoglycemia. An oral glucose tolerance test was normal. Fasting hypoglycemia was diagnosed after she underwent a 72-hour fast resulting in a low serum glucose of 3.1, with simultaneous inappropriately high insulin of 150 (NR < 95 pmol/L) and C-peptide at 1301 (NR: 325-1090). Imaging was then performed to try and determine the cause of her hypoglycemia. CT and MRI scans did not identify any pancreatic lesions or insulinomas, and an octreotide study did not show any areas of increased uptake. The patient's symptoms intensified, with nightly hypoglycemic symptoms that resulted in an inability to work. The patient's symptoms could not be controlled with medical management, so further investigations were undertaken in the hope of finding a lesion that could be surgically treated.

Selective arterial calcium stimulation (SACST) with hepatic venous sampling was performed, using the methods described by Thompson et al. [4]. There was a significant relative elevation in insulin levels following the stimulation of the superior mesenteric (SMA), gastroduodenal (GDA), and splenic arteries (SA), as shown in Table 1. No elevation was seen from the stimulation of the gastroepiploic (GEA) and hepatic arteries (HA). The maximal insulin concentration was seen in response to the stimulation of the SMA, at 235 pmol/L. The response from 3 arteries in SACST strongly suggested the presence of diffuse nesidioblastosis instead of a very small insulinoma [4], and the response from the SA suggested that the nesidioblastosis would be located around the tail of the pancreas [5]. However, the lack of response from 2 arteries suggested that the nesidioblastosis did not extend through the entire pancreas.

**Figure 1:**
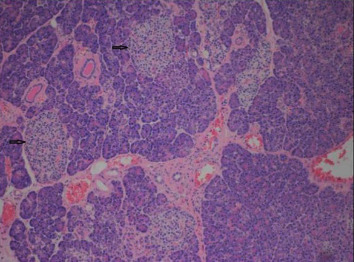
Pancreatic parenchyma at 100× magnification, with arrows indicating enlarged islets.

**Figure 2:**
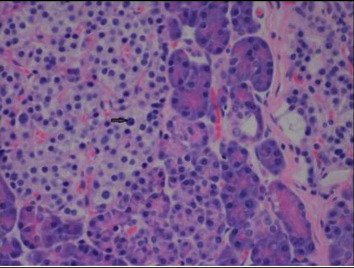
Islet cells at 200× magnification, with arrow indicating an enlarged nucleus.

On the basis of the SACST results, a distal pancreatectomy was performed, removing the pancreatic tail region measuring 14.5 × 5.2 × 2.5 cm, and preserving the spleen and duodenum. The histopathology confirmed the presence of diffuse nesidioblastosis within the pancreatic tail, with increased numbers of islet cells (Figure 1), some of which had enlarged nuclei (Figure 2). Furthermore, when sectioned there was no evidence of discrete masses or lesions. This pathology was found to be uniformly distributed throughout all of the resected surgical specimens. While the remaining pancreas was not biopsied to prove the absence of pathological features of nesidioblastosis, we assume that there was no significant amount of remaining abnormality due to the elimination of hypoglycemia immediately postoperation.

The patient has done well postoperatively with no further hypoglycemia and normal fasting glucose tolerance for 34 months.

## 2. Discussion

The differential diagnosis of endogenous hyperinsulinemic hypoglycemia in adults includes insulinoma and adult-onset nesidioblastosis. Insulinoma is significantly more common than nesidioblastosis, with approximately 1 to 3 cases per million [6]. Nesidioblastosis is estimated to account for 1% to 5% of cases of adult endogenous hyperinsulinemic hypoglycemia [7]. Clinically and biochemically, nesidioblastosis cannot be distinguished from insulinomas, and insulinomas smaller than 1 cm cannot be detected using imaging techniques [8], further complicating the differential diagnosis.

Selective arterial calcium stimulation can be used to aid in distinguishing between insulinoma and diffuse nesidioblastosis; if there is a significant relative elevation in insulin with the stimulation of 2 or more arteries, then nesidioblastosis is the probable diagnosis [4]. This is likely due to the diffuse nature of nesidioblastosis, whereas insulinomas are more localized and are more likely to respond only to stimulation of one artery. This also allows for some localization of nesidioblastosis, as in this case, where arterial stimulation aided in determining that the nesidioblastosis was primarily located in the pancreatic tail. However, there is some challenge due to individual variations in pancreatic arterial perfusion and anatomy that may produce problems when attempting to localize the abnormal cells [9].

Nesidioblastosis is characterized by the presence of many beta cells with enlarged nuclei and large amounts of clear cytoplasm, in most islets throughout the pancreas [10]. Typically, no abnormalities are seen in somatostatin, glucagon, and pancreatic polypeptide cells [7, 11]. Some patients have increased numbers of beta cells and islet hypertrophy, but this is not seen in all cases [12]. Peliosis-like vascular ectasia has been seen in some cases, often when other endocrinopathies are involved [13, 14]. Increased numbers of ductuloinsular complexes are seen occasionally, although most cases lack this feature [7]. Our case did show some beta cells with enlarged nuclei, and there were also an increased number of beta cells with islet hypertrophy. However, there was not an increase in ductuloinsular complexes, and no peliosis-like vascular ectasia was noted.

Diffuse nesidioblastosis is by far the most common form in adults [7]. Rare cases of adult focal nesidioblastosis have been reported [15–17], wherein exophytic lesions have been found on the pancreas containing cells characteristic of nesidioblastosis. Focal nesidioblastosis is much more common in neonates, making up roughly 40% of congenital nesidioblastosis cases [18].

In focal nesidioblastosis, abnormal beta cells are confined to exophytic lesions on the surface of the pancreas, whereas diffuse nesidioblastosis typically involves the entire pancreas [3].

We believe that our case represents a form of nesidioblastosis that is intermediate between focal and diffuse nesidioblastosis, as it was functionally localized to the tail region, but was clearly spread throughout the pancreas and was not exophytic. The localization is evidenced by our patient's lack of hypoglycemia after distal pancreatectomy, which is fairly rare when compared to other cases.

As shown in Table 2, most patients that undergo distal pancreatectomy for nesidioblastosis have further hypoglycemia, often requiring medication. This suggests that in their cases, the defective beta cells are spread throughout the entire pancreas. Even when subtotal pancreatectomy is considered, over half of patients still experience hypoglycemia. Furthermore, in cases where near-total or completion pancreatectomy was performed, nesidioblastosis was found throughout the entire pancreas [26].

We believe that we report the first case of adult nesidioblastosis that is diffuse in nature but limited to a specific region of the pancreas. It supports the value of using SACST in the diagnostic evaluation of patients with fasting hypoglycemia who do not have a mass visible on imaging. Knowledge of this condition will help plan the surgical approach and also provide physicians and patients a realistic expectation of the likelihood that partial pancreatic resection will reduce hypoglycemia to a meaningful degree.

## Figures and Tables

**Table 1:** Insulin concentrations (pmol/L) measured during SACST, at baseline levels, and then 20, 40, and 60 seconds after calcium gluconate injection.

	SMA	GDA	GEA	HA	SA
Baseline	28	28	24	29	24
20 sec.	26	83	27	40	30
40 sec.	235	85	33	33	73
60 sec.	156	71	31	32	58

**Table 2:** Selected previously reported cases of idiopathic nesidioblastosis with follow-up after surgery [7, 11, 19–27]. Distal pancreatectomy refers to the removal of the body and tail of the pancreas, typically no more than 60%. Subtotal pancreatectomy refers to removal of approximately 80% of the pancreas.

	Distal pancreatectomy	Subtotal pancreatectomy
Recurring hypoglycemia	12	3
No hypoglycemia	7	2

## Data Availability

Data from Table 2 can be accessed from the articles cited.
